# Case-finding for common mental disorders of anxiety and depression in primary care: an external validation of routinely collected data

**DOI:** 10.1186/s12911-016-0274-7

**Published:** 2016-03-15

**Authors:** Ann John, Joanne McGregor, David Fone, Frank Dunstan, Rosie Cornish, Ronan A. Lyons, Keith R. Lloyd

**Affiliations:** Farr Institute of Health Informatics Research, Swansea University Medical School, Singleton Park, Swansea, SA2 8PP UK; Public Health Wales NHS Trust, Cardiff, UK; Institute of Primary Care and Public Health, School of Medicine, Cardiff University, Cardiff, CF14 4YS UK; School of Social and Community Medicine, University of Bristol, Oakfield House, Oakfield Grove, Bristol BS8 2BN UK

**Keywords:** Anxiety, Depression, Electronic health records, Validation

## Abstract

**Background:**

The robustness of epidemiological research using routinely collected primary care electronic data to support policy and practice for common mental disorders (CMD) anxiety and depression would be greatly enhanced by appropriate validation of diagnostic codes and algorithms for data extraction. We aimed to create a robust research platform for CMD using population-based, routinely collected primary care electronic data.

**Methods:**

We developed a set of Read code lists (diagnosis, symptoms, treatments) for the identification of anxiety and depression in the General Practice Database (GPD) within the Secure Anonymised Information Linkage Databank at Swansea University, and assessed 12 algorithms for Read codes to define cases according to various criteria. Annual incidence rates were calculated per 1000 person years at risk (PYAR) to assess recording practice for these CMD between January 1^st^ 2000 and December 31^st^ 2009. We anonymously linked the 2799 MHI-5 Caerphilly Health and Social Needs Survey (CHSNS) respondents aged 18 to 74 years to their routinely collected GP data in SAIL. We estimated the sensitivity, specificity and positive predictive value of the various algorithms using the MHI-5 as the gold standard.

**Results:**

The incidence of combined depression/anxiety diagnoses remained stable over the ten-year period in a population of over 500,000 but symptoms increased from 6.5 to 20.7 per 1000 PYAR. A ‘historical’ GP diagnosis for depression/anxiety currently treated plus a current diagnosis (treated or untreated) resulted in a specificity of 0.96, sensitivity 0.29 and PPV 0.76. Adding current symptom codes improved sensitivity (0.32) with a marginal effect on specificity (0.95) and PPV (0.74).

**Conclusions:**

We have developed an algorithm with a high specificity and PPV of detecting cases of anxiety and depression from routine GP data that incorporates symptom codes to reflect GP coding behaviour. We have demonstrated that using diagnosis and current treatment alone to identify cases for depression and anxiety using routinely collected primary care data will miss a number of true cases given changes in GP recording behaviour. The Read code lists plus the developed algorithms will be applicable to other routinely collected primary care datasets, creating a platform for future e-cohort research into these conditions.

**Electronic supplementary material:**

The online version of this article (doi:10.1186/s12911-016-0274-7) contains supplementary material, which is available to authorized users.

## Background

Common mental disorders (CMD) are an important public health problem comprising depression, anxiety, panic and somatisation and usually presenting as mixed syndromes with mixed symptoms. They are significant contributors to impaired health, well-being and health services utilisation, direct and indirect costs to the economy and overall mortality [1–4] CMD have a combined community prevalence of between 15 % and 30 %, depending on the population and case definition used [5, 6].

The community prevalence of CMD is significantly greater than in primary are because only around one-third of affected people seek help in primary care [7]. Among attendees, depression and anxiety (the most common CMDs) often go unrecognised [7–11]. Ten years ago, of those recognised, fewer than one-third received treatment [12]. Up to three-quarters may have visited their physician in the previous year with seemingly unrelated complaints [13–16]. In primary care settings, decisions about who should receive treatment for recognised CMD seem to be made on a patient-by-patient basis, influenced by the severity of symptoms demonstrated [17]. In the UK over the last decade, GPs have become more likely to record individual symptoms rather than specific CMD diagnoses [18, 19].

Routinely collected electronic health records have the potential to support policy and enhance the practice of health and social care. The validity and reliability of research using routinely collected primary care electronic data depends upon its quality and completeness. Overall, the validity of primary care diagnoses in the UK tends to be high, although the quality of reporting and detailed descriptions of validation methods are often variable and inadequate [20].

Any method clarifying the most robust way to identify a case of anxiety and depression from routinely collected primary care data would support research in this area. Previously we identified suitable participants for a trial in depression using electronic general practice data by an internal validation methodology involving two independent clinicians [21]. In this study we assess our ability to identify cases of CMD in the primary care dataset by an external validation method using a survey administered mental health score.

## Methods

### Aim

We aimed to create a robust research platform for CMD (anxiety and depression) using population-based, routinely collected primary care electronic data.

### Objectives

We planned to create Read code lists and algorithms to identify primary care recorded CMD anxiety and depression in order to assess any changes in diagnostic recording for CMD anxiety and depression in the General Practice Dataset (GPD) between January 1^st^ 2000 and December 31^st^ 2009 in those aged over 18 years. These algorithms for case-finding of primary care recorded anxiety and depression will then be compared to the five-item Mental Health Inventory (MHI-5), as a measure of mental health status.

### Ethical approval

The ethical considerations of this project were covered by permissions granted to the Swansea Secure Anonymised Information Linkage (SAIL) Databank and the Caerphilly Health and Social Needs Survey (CHSNS) [22]. Additionally we gained approval from the Swansea University Information Governance Review Panel, an independent body consisting of a range of government, regulatory and professional agencies, including the NHS Research Ethics Service and members of the public, which grants approval to studies conducted within the SAIL Databank.

### Data source

#### Caerphilly health & social needs survey

The CHSNS is a community study of health inequality set in Caerphilly county borough, Wales [22–24]. The current dataset comprises a two-wave cross-sectional postal questionnaire survey. The baseline survey sample (wave 1) was drawn from a total population of 132,613 residents aged 18 years and over recorded in the then current local General Medical Practice administrative register on May 31^st^ 2001. This produced a representative dataset on 10,892 residents of the borough, aged 18 to 74 years. The response rate for wave 1 was 63.0 %. In 2008, a follow-up postal questionnaire survey was carried out (wave 2) of surviving baseline respondents, then aged 25 to 81 years, with a response rate of 50.2 % [23, 24].

### Measure of mental health

Both waves of the survey included responses to SF-36, version 2, health status questionnaire. The measure of mental health in this study was the five-item MHI-5, a sub-scale of the SF-36. Its validity and reliability, as a measure of mental health status, are well established [25, 26] and reflect the continuously distributed, single dimensional nature of CMD symptoms in the population [27, 28]. We restricted this analysis to those aged under 75 years at baseline, the MHI-5 being less reliable in UK elderly populations [29, 30]. The assessment of CMD using the MHI-5 scale has been performed previously and is well established [22, 31–34]. It performs at least as well as the General Health Questionnaire [22, 31–36]. The response scores were transformed using the scale developers’ published method, with imputation of missing data, to a scale of range 0 to 100, with lower scores indicating poorer mental health [37].

Our previous work [38] has demonstrated a range of methods for producing a cut-point on the MHI-5 thus overcoming earlier limitations [31, 39]. We state that investigators should consider carefully the cut-point most suitable for their study based primarily on the intended application of the resulting cut-point. We chose a cut-point of ≤60 on the MHI-5 to minimise misclassification rate since we were interested in the within-borough comparisons between MHI-5 scores and GP diagnoses.

### SAIL Databank

**Fig. 1:**
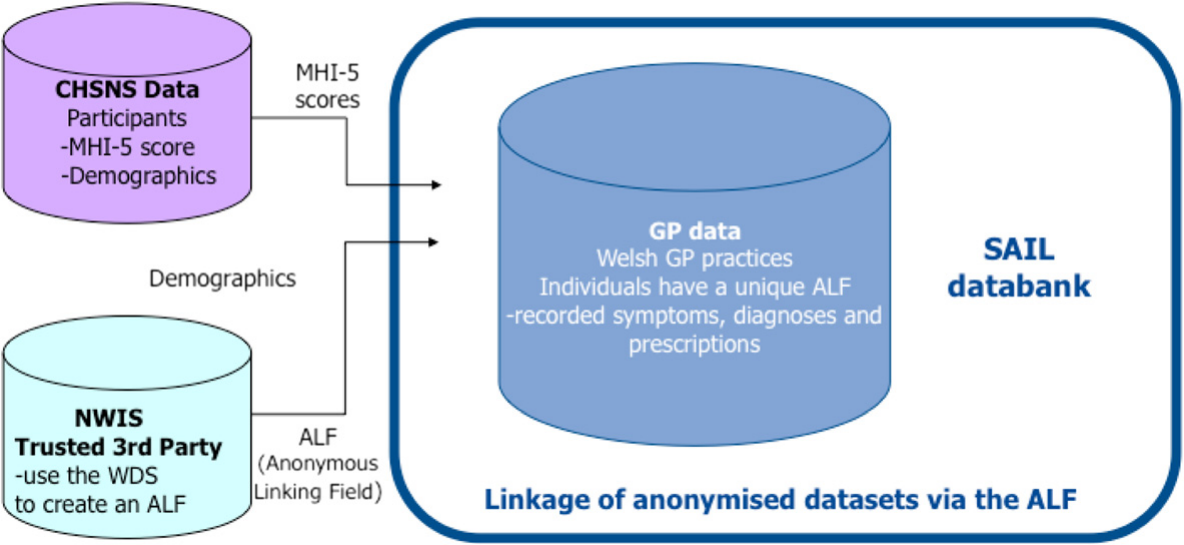
Split file approach to linking survey data to primary care data

The SAIL databank is the national data repository of anonymised, person-based, linkable data in Wales covering a population of 3 million [40, 41]. Robust policies, structures and controls are in place to protect privacy through a reliable matching, anonymisation and encryption process achieved in conjunction with the NHS Wales Informatics Service (NWIS) using a split file approach as detailed by Ford et al. (2009) and Lyons et al., (2009). The split file approach involves separation of identifiers from clinical content, identity matching and creation of pseudonymised linkage keys (Anonymised Linking Fields) by NWIS prior to reassembling and further encryption of data sets using different algorithms within the University (Fig. 1).

The primary care dataset in SAIL (GPD) contains Read codes for each registered individual in a SAIL supplying practice. Read codes are a hierarchical nomenclature used to record clinical summary information. Primary care physicians enter medical diagnoses and symptoms using Read codes. The GPD does not contain any accompanying free text on referral or discharge to or from secondary/tertiary care. It is regularly updated.

### E-cohort creation

The survey dataset was imported into the SAIL databank and an electronic cohort created by record-linking the baseline survey data demographics to the primary care dataset using the Welsh Demographic Service (WDS) dataset in NWIS. WDS contains the unique NHS number for all individuals who register with the free to use general medical practitioner service (Fig. 1).

### Primary care case selection

Read code lists, corresponding to ICD-10 Chapter V [42] diagnoses of anxiety and depression, were developed by clinical members of the study team with reference to Rait et al. [18] and the Quality Outcomes Framework [43] (Additional file 1: Table S1). These included GP recording of i) anxiety diagnoses e.g. generalized anxiety disorder; ii) anxiety symptoms e.g. anxiousness; iii) mixed anxiety and depression; iv) panic attacks and panic disorders; v) depression diagnoses; vi) depression symptoms. We excluded codes for other psychosis, phobias, obsessive compulsive disorders, post traumatic stress disorder, behavioural disorders, hyperkinetic disorders, conduct disorders and disorders of social functioning in keeping with other studies of this type [18, 19]. We excluded adjustment disorders as conceptually they are an intermediate health condition between normal responses to stress and more severe emotional disorders such as anxiety and depression [44]. We also compiled a Read code list of British National Formulary listed antidepressants, anxiolytics and hypnotics [45] (Additional file 2: Table S2).

We queried the GPD using db2 structured query language, implementing Read Codes Version 2 (5-byte set). We used, devised algorithms and evaluated multiple methods to define a case of anxiety and depression (12 in total as listed in Table 2) incorporating, in various combinations, current and historical diagnoses, symptoms and treatments. Our definition of ‘current’ was a search for relevant Read codes over a one-year period with the date of the survey response at the midpoint. This was in order to capture those who may present to their GP with CMD but not be diagnosed for a period of time and also those who may delay seeing their GPs for a period of time. Our definition of ‘historical’ was a search for relevant Read codes through the retrospective longitudinal data housed in the GPD outside the ‘current’ period. The length of retrospective data varied between individuals depending on the length of their registration with a SAIL supplying practice and was longer for wave 2 respondents. Treatment was at least one prescription for an antidepressant, anxiolytic or hypnotic in the one-year current period.

### Statistical analysis

The data were analysed using SPSS Statistics software (Version 20). We calculated a ten-year period prevalence of anxiety and depression diagnosis in the GPD population between January 1^st^ 2000 and December 31^st^ 2009 in those aged over 18 years using the study Read code lists. We calculated annual incidence rates of recorded anxiety and depression, based on diagnoses, symptoms and diagnoses and symptoms combined, between 2000 and 2010 to assess changes in diagnostic recording practice for the inclusion of certain codes in our study. A new episode was defined as an entry in the records with no previous entry of that problem recorded in the previous year. We therefore reviewed data from January 1^st^ 1999.

Annual incidence rates were calculated per 1000 person years at risk (PYAR). We calculated person-time at risk using the start of each year (1^st^ January) or start of registration (plus 6 months) whichever was the later for each of the years. The end date was the earliest of the following: date of leaving a SAIL supplying practice, date of death or the end of the target year (31^st^ December). We excluded the first six months of data following registration. This allowed for retrospective recording to reduce the chance of prevalent cases being recorded as incident. It was a requirement that each individual contributed at least one year’s follow-up data.

We then compared the ‘primary care cases’, for each of the 12 definitions, to the ‘survey cases’ separately for both waves of the survey. Sensitivity, specificity and positive predictive values (PPV) for the 12 defined algorithms were calculated, using a cut-point of ≤60 for the MHI-5 (as gold standard) [38]. Sensitivity measures the proportion of cases and specificity the proportion of non-cases, identified in the survey data, correctly identified as such in primary care. The PPV is the probability that the person identified by the algorithm is a survey case and is expressed as a proportion.

We explored the reasons for algorithm 9, based on diagnosis and treatment codes only, identifying false negatives and false positive cases (including the role of symptom codes in this) in the Wave 2 sample since these are the codes used in current literature.

## Results

### Identification of READ codes corresponding to anxiety and depression diagnosis in the SAIL databank GPD

Five hundred twenty two thousand and five hundred seventy eight patients were registered continuously with a GP within the SAIL databank from 1^st^ January 2000 to 31^st^ December 2009. The ten-year period prevalence of GP recorded diagnoses of anxiety and depression during this time was 16.2 % (*n* = 84,835). Where individuals had more than one type of diagnosis, the ten-year period prevalence for depressive disorders was 9.3 % (*n* = 48,382), mixed anxiety and depression 4.8 % (*n* = 25,256), and anxiety disorders 6.4 % (*n* = 33,284). If depression and anxiety symptom codes were included, the ten-year prevalence was 21.4 % (*n* = 111,768).

### GPs’ electronic recording of depression and anxiety

**Fig. 2:**
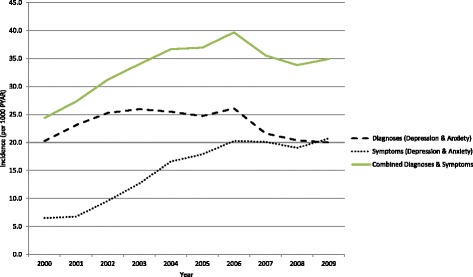
Trends in the incidence of GP recorded anxiety and depression (2000–2010)

Table 1 and Fig. 2 show trends in the incidence of depression and anxiety between 1^st^ January 2000 and 31^st^ December 2009. The incidence of recorded depression and anxiety diagnoses (combined) remained stable over the ten-year period but the incidence of recorded symptoms increased substantially from 6.5 to 20.7 per 1000 PYAR. The combined incidence of diagnoses and symptoms of depression and anxiety increased from 24.4 to 34.9 1000 PYAR.

**Table 1:** Incidence of GP recorded depression/anxiety broken down by diagnoses and symptoms in the SAIL databank 2000–2010 per 1000 person-years at risk (PYAR)

Year	Diagnosis recorded (Depression & Anxiety)	Symptom recorded (Depression & Anxiety)	Either Diagnosis or symptom recorded
2000	20.3	6.5	24.4
2001	23.1	6.8	27.3
2002	25.3	9.6	31.2
2003	26.0	12.7	34.0
2004	25.5	16.6	36.7
2005	24.7	17.9	37.0
2006	26.1	20.3	39.7
2007	21.6	20.1	35.6
2008	20.4	19.0	33.8
2009	20.0	20.7	34.9

### Study population

There were 691,762 individuals aged 18–74 (38.5 % of relevant population in Wales) registered to a SAIL supplying practice continuously between 1^st^ May 2001 and 30^th^ April 2002 (wave 1 period) and 805,929 (44.8 % of relevant population in Wales) individuals aged 18–74 registered continuously to a SAIL supplying practice between 1^st^ May 2008 and 30^th^ April 2009 (wave 2 period).

The CHSNS dataset included 10,653 MHI-5 scores from wave 1 and 4,426 MHI-5 scores from wave 2. We matched 2,584 of 10,653 (24.3 %) wave 1 survey responders and 1,195 of 4,426 (27.0 %) wave 2 responders, corresponding to 2799 individuals with MHI-5 scores, to their primary care data. This was on the basis of continuous registration with a SAIL supplying practice for the 12-month period at the time of the survey response. Among these, 824 (32 %) had scores of ≤60 on the MHI-5 at Wave 1 and 395 (33 %) at Wave 2.

There were 84 (3.3 %) CHSNS MHI-5 responders in wave 1 and 54 (4.5 %) in wave 2 linked in SAIL with a current diagnosis or symptom of anxiety and depression in the GPD. There were 198 (7.7 %) responders in wave 1 and 188 (15.7 %) in wave 2 linked in SAIL with a historical diagnosis or symptom of anxiety or depression currently treated in GPD.

### Validation results

Table 2 shows the sensitivity, specificity and PPV for the comparison of GP Read codes for anxiety and depression diagnoses, symptoms and treatment with MHI-5 data from the CHSNS waves using a cut-point of ≤60. As expected, all the algorithms underestimated the prevalence of CMD compared to the MHI-5, particularly those based only on current diagnosis or symptoms. The addition of historical diagnoses with current treatment contributed most to increasing the sensitivity.

**Table 2:** Sensitivity, specificity, positive predictive value-results comparing GP Read codes for anxiety and depression diagnoses, symptoms and treatment with MHI-5 data (as the gold standard) from the CHSNS waves

Algorithm: CMD diagnosis/symptom/treatment	Wave (W)	True Positives^a^	True negatives^b^	Sensitivity	Specificity	Positive Predictive value	Number of cases identified by algorithm n (prevalence, %)–includes false positives W1, *n* = 2584 W2, *n* = 1195
1. Current treatment only	W1	268	1590	0.33	0.90	0.61	438 (17.0)
	W2	189	712	0.48	0.89	0.68	277 (23.2)
2. Current diagnosis (treated or untreated)	W1	50	1737	0.06	0.99	0.68	73 (2.8)
	W2	20	792	0.05	0.99	0.71	28 (2.3)
3. Current diagnosis treated	W1	48	1742	0.06	0.99	0.73	66 (2.6)
	W2	18	793	0.05	0.99	0.72	25 (2.1)
4. Current diagnosis or symptom (treated or untreated)	W1	57	1733	0.07	0.99	0.68	84 (3.3)
	W2	37	783	0.09	0.98	0.69	54 (4.5)
5. Current diagnosis or symptom (treated only)	W1	53	1738	0.06	0.99	0.71	75 (2.9)
	W2	32	785	0.08	0.98	0.68	47 (3.9)
6. Current treatment plus untreated current diagnosis or symptom	W1	272	1585	0.33	0.90	0.61	447 (17.3)
	W2	194	710	0.49	0.89	0.68	284 (23.8)
7. Historical or current diagnosis currently treated	W1	121	1697	0.15	0.96	0.66	184 (7.1)
	W2	113	765	0.29	0.96	0.76	148 (12.4)
8. Historical or current diagnosis or symptom currently treated	W1	131	1693	0.16	0.96	0.67	198 (7.7)
	W2	138	750	0.35	0.94	0.73	188 (15.7)
9. Historical diagnosis currently treated plus current diagnosis (treated or untreated)	W1	123	1692	0.15	0.96	0.64	191 (7.4)
	W2	115	764	0.29	0.96	0.76	151 (12.6)
10. Historical diagnosis currently treated plus current diagnosis or symptom (treated or untreated)	W1	127	1691	0.16	0.96	0.65	196 (7.6)
	W2	126	756	0.32	0.95	0.74	170 (14.2)
11. Historical or current diagnosis or symptom currently treated plus current diagnosis untreated	W1	133	1688	0.16	0.96	0.65	205 (7.9)
	W2	140	749	0.35	0.94	0.73	191 (16.0)
12. Historical or current diagnosis or symptom currently treated plus current diagnosis or symptom untreated	W1	135	1688	0.17	0.96	0.65	207 (8.0)
	W2	143	748	0.36	0.94	0.73	195 (16.3)

^a^Number identified with CMD using MHI-5 data at Wave 1 = 824, at Wave 2 = 395

^b^Number identified as not having CMD at Wave 1 = 1760, at Wave 2 = 800

### False positives

Based on algorithm 9 (historical diagnosis currently treated plus current diagnosis treated or untreated) using wave 2 data we incorrectly identified 36 of 800 patients (4.5 %) as a case of CMD in SAIL, all of whom had at least one historical code for a diagnosis of depression and/or anxiety (Table 3). Of these, 35 (97.2 %) were being currently treated.

**Table 3:** False positives and false negatives for Algorithm 9

	MHI-5 CMD case	MHI-5 no CMD	Totals
GPD case	115	36	151
GPD no CMD	280	764	1044
Totals	395	800	1195

### False negatives

A total of 280 of 395 (70.9 %) survey cases were not identified using this algorithm (Table 3). Of these, 76 (27.1 %) were currently being treated but had no diagnostic Read codes for anxiety or depression recorded in their GP records. However, 25 (8.9 %) individuals did have current symptom codes, and 32 (11.4 %) had historical diagnosis codes (17 of whom had a historical symptom code), but were not being currently treated and nine (3.2 %) were registered with a practice but had no record of any attendances with their GP during that one-year period.

The majority of false negatives 200/280 (71 %) had a historical recording of pain and 139/280 (50 %) had one of the following chronic physical diseases–cardiovascular disease, diabetes, digestive disorder, kidney disorder, thyroid problem, chronic migraines, chronic obstructive pulmonary disease or one of the top seven cancers (as defined by World Health Organisation). Thirty-nine of the 280 (14 %) subjects had CMD other than depression and anxiety such as stress or other neurotic disorders. Alcohol dependency accounted for 6.8 % (19/280). Other mental health related disorders such as psychotic disorders and drug dependencies were present; however, they accounted for a very small number.

## Discussion

### Main findings

We have created a set of algorithms to identify cases of CMD anxiety and depression from routinely collected GP data. We linked survey data containing a validated instrument (MHI-5) for CMD to the GP record and compared results with recorded anxiety and depression diagnoses, symptoms and treatment codes to assess the sensitivity, specificity and PPV of the group of codes and algorithms chosen.

We then calculated the ten-year period prevalence and incidence of anxiety and depression diagnosis in a large primary care population. The recorded incidence of combined anxiety and depression diagnoses in Wales has remained stable over time while the incidence of symptoms has increased. We therefore included symptom codes in our analysis.

We found that using algorithm 9 (a historical diagnosis currently treated plus a current diagnosis whether treated or untreated) at wave 2 resulted in a specificity of 0.96, sensitivity 0.29 and PPV 0.76. This method improves sensitivity considerably from using current diagnosis alone, as is done in many studies using routine data, whilst specificity remains high. It had the optimal combination of specificity and PPV.

All false positive cases had historical diagnoses and were currently treated. These may be cases that were benefiting from their treatment and were asymptomatic at the time of survey. False negatives, i.e. those identified in the survey as having anxiety and depression but not identified in primary care, were mainly those with historical diagnoses of pain (71 %) or who had chronic co-morbidities (50 %). Approximately 9 % of false negatives had current symptom codes and 14 % had CMD other than depression and anxiety recorded in primary care. The latter is a limitation of using the MHI-scale in its assessment of mental health status.

The MHI-5 scale asks questions relating to current status (previous four weeks) and therefore current symptoms will play an important role in questionnaire completion. Additionally there appears to be an increasing preference over time for recording symptoms over diagnoses in primary care [18, 19]. Adding current symptom codes to the algorithm improved sensitivity (0.32) with a marginal effect on specificity (0.95) and PPV (0.74). This algorithm (algorithm 10) produces a sample size for analysis that is over five times that where current diagnosis (algorithm 2) alone is used to identify cases (Table 2).

### Strengths and limitations

The assessment of CMD using MHI-5 has been made previously [31, 36], although not through data linkage. However the gold standard for identifying cases would be to use the Clinical Interview Schedule-Revised [46]. A strength of this study is the analysis of large population level data giving large sample sizes for calculating the incidence of recorded anxiety and depression. The use of routinely available data minimises the costs of epidemiological research. The linkage of survey data to SAIL data allowed for the validation of Read codes and the use of these Read codes and algorithms in further epidemiological research [47].

Response bias is a potential issue in the CHSNS. The response rate to the first wave of the CHSNS is comparable to many population surveys, but the wave 2 response was lower at 50.2 %. Selection bias is a potential issue in using the GPD. The GPD population studied is large but covers 168 out of 474 practices in Wales and 38.5 % (wave 1 period)/44.8 % (wave 2 period) of the population aged 18–74 years. Those practices that are not currently signed up to SAIL may be in some way different to those that are. This may introduce bias in the estimation of prevalence. However, it is unlikely that the relationship between GP-recorded CMD and CMD measured using the MHI-5 would be different among practices in SAIL and not in SAIL. As such our estimates of the relative performance of the different algorithms are unlikely to have been affected. The sensitivity of our case definitions is higher in wave 2 than wave 1 since more historical data are available and also, possibly, because of the increase in the number of prescriptions issued for antidepressants, anxiolytics and hypnotics over the last decade [18].

A further strength of this study is the inclusion of symptom codes. A recent study [48] found that CMD and sub threshold psychiatric symptoms were both independently associated with new-onset functional disability and significant days lost from work. They suggest leaving symptoms unaccounted for in surveys may lead to gross underestimation of disability related to psychiatric morbidity.

Given the changing patterns of coding behaviour by GPs and the constantly evolving Read code system, assessment of prevalence based on these data alone would be flawed. Analysis of GP utilisation data extrapolates an estimate of community demand and need based upon existing utilisation experience. It says more about mental health resources, practice priorities, help-seeking behaviour and service capacity than about the real prevalence or need in the population. Because we have been able to externally validate these codes using the MHI-5, they can be used as a platform for epidemiological research into the CMD anxiety and depression and as an outcome measure using routinely available GP data for various studies including electronically linking participants in trials and traditional cohorts.

For epidemiological research, using large computerised databases of routinely collected medical records, robust findings rely on validation of methods for case ascertainment. The method of identifying a case of anxiety and depression from routinely collected primary care data, the trade-off between sensitivity and specificity and hence the choice of algorithm, will vary with study design. High specificity and therefore PPV, if necessary at the expense of low sensitivity, is more crucial for validity in creating primary care e-cohorts so that most cases identified have the disorder of interest. This is particularly important for case control studies, a common design in e-studies where identification of large numbers of controls is facilitated. Where the ratio of controls to cases is high, misclassification of cases as controls, i.e. a high false negative rate, may not bias results significantly. However, misclassifying controls as cases has the potential for bias. In such studies high PPVs and specificity are important. We have explored how different combinations of codes and algorithms affect these measures allowing for a more robust understanding when using routine data. For example using the algorithm based on current diagnosis alone is highly specific and comparable with current literature. However the sensitivity is low and the sample size in this study would be small.

### Comparison with previous literature

The prevalence of the CMDs of anxiety and depression found in this study is in keeping with other studies [4, 6, 9]. Changes in primary care recording of anxiety and depression in Wales mirror those found in other large primary care datasets, [18, 19] and justify the inclusion of symptom codes in our analysis rather than reflecting a true decrease in the incidence of the diagnosis of anxiety and depression. Strategies adopted in the Quality and Outcomes framework for those with diagnosed depression may be having unintended consequences on coding patterns possibly resulting in a disincentive to record diagnoses [49].

Based on an MHI-5 cut point of ≤60, GPs diagnosed around 30 % of participants defined as a case of CMD by the MHI-5 score as having anxiety or depression (diagnosis or symptom). This is similar to findings in the Dutch population [39]. Several explanations exist why those with probable CMD do not seek healthcare or, if they do, why the general practitioner does not diagnose them. These include stigma, spontaneous resolution and patients presenting with physical symptoms/problems [47].

### What this study adds

We have developed an algorithm with excellent specificity and high PPV for detecting CMD (anxiety and depression) with a trade off of low but expected sensitivity from routinely collected electronic primary care data for research purposes. The Read code (diagnosis, symptoms and treatment) lists plus the developed algorithms will be applicable to other primary care datasets of routinely collected data, thus creating a platform for future e-cohort research into these conditions. We have demonstrated that using diagnosis and current treatment alone to identify cases for depression and anxiety using routinely collected primary care data will miss a number of true cases given changes in GP recording behaviour. We have also shown that the algorithms are enhanced by the inclusion of current symptoms.

### Implications

De-identified databanks of routinely collected clinical data such as SAIL, CPRD (Clinical Practice Research Database) [50] and THIN (The Health Improvement Network) [51] [provide a rich resource for research. The development of algorithms from complex datasets that identify cases with high PPV is an important step in epidemiological research. This work has implications for future research on the common mental disorders anxiety and depression, and on antidepressants. By understanding the performance of the different algorithms we gain a lot of insight into their potential use for research. We are now including the CMD as mental health outcomes in studies across a range of areas, including the environment, housing, suicide and for clinical research [47, 52–54]. We plan to externally validate the algorithms developed to assess CMD in children and young people using age-appropriate survey data.

## Conclusions

The assessment of cases of CMD (anxiety and depression) based on those with a historical diagnosis currently treated plus a current diagnosis or symptom treated or untreated (algorithm 10) will be useful as a platform for future research in e-cohort studies using routinely collected primary care data. Assessing diagnosis only, in addition, would allow comparison with other literature.

### Availability of data and materials

Data will not be shared due to permissions granted under the SAIL databank.

## Additional files

Additional file 1: Depression and Anxiety Read Codes V2 used in algorithms. (DOCX 16 kb) Additional file 2: Drug treatment Read Codes V2 used in algorithms. (DOCX 16 kb)

## Abbreviations

CHSNS: caerphilly health and social needs survey
CMD: common mental disorder
CPRD: clinical practice research database
GPD: general practice dataset
MHI-5: mental health inventory (sub-scale of SF-36)
PPV: positive predictive value
PYAR: person years at risk
SAIL: secure anonymised information linkage
SF-36: short form health status questionnaire
THIN: the health improvement network
WDS: welsh demographic service
