# Supplementary material for: Case-finding for common mental disorders of anxiety and depression in primary care: an external validation of routinely collected data
Source: BMC Med Inform Decis Mak. 2016 Mar 15;16:35. doi: 10.1186/s12911-016-0274-7 (PMC4791907; doi:10.1186/s12911-016-0274-7)
Supplement: Additional file 1: — Depression and Anxiety Read Codes V2 used in algorithms. (DOCX 16 kb) [file 12911_2016_274_MOESM1_ESM.docx]

**Depression and Anxiety Read Codes V2 used in algorithm**

***Depression Diagnoses***

Eu32. [X]Depressive episode

Eu320 [X]Mild depressive episode

Eu321 [X]Moderate depressive episode

Eu322 [X]Severe depressive episode without psychotic symptoms

Eu324 [X]Mild depression

Eu32y [X]Other depressive episodes

Eu32z [X]Depressive episode, unspecified

Eu33. [X]Recurrent depressive disorder

Eu330 [X]Recurrent depressive disorder, current episode mild

Eu331 [X]Recurrent depressive disorder, current episode moderate

Eu332 [X]Recurrent depressive disorder, current episode severe without psychotic symptoms

Eu334 [X]Recurrent depressive disorder, currently in remission

Eu33y [X]Other recurrent depressive disorders

Eu33z [X]Recurrent depressive disorder, unspecified

Eu341 [X]Dysthymia

E118. Seasonal affective disorder

E135. Agitated depression

E2B.. Depressive disorder NEC

E2B1. Chronic depression

E291. Prolonged depressive reaction

E204. Neurotic depression reactive type

E2B0. Postviral depression

E112. Single major depressive episode

E1120 Single major depressive episode, unspecified

E1121 Single major depressive episode, mild

E1122 Single major depressive episode, moderate

E1123 Single major depressive episode, severe, without psychosis

E1125 Single major depressive episode, partial or unspecied remission

E1126 Single major depressive episode, in full remission

E112z Single major depressive episode NOS

E113. Recurrent major depressive episode

E1130 Recurrent major depressive episodes, unspecified

E1131 Recurrent major depressive episodes, mild

E1132 Recurrent major depressive episodes, moderate

E1133 Recurrent major depressive episodes, severe, no psychosis

E1135 Recurrent major depressive episodes, partial/unspecified remission

E1136 Recurrent major depressive episodes, in full remission

E1137 Recurrent depression

E113z Recurrent major depressive episode NOS

***Anxiety diagnoses***

Eu41. [X]Other anxiety disorders

Eu410 [X]Panic disorder [episodic paroxysmal anxiety]

Eu411 [X]Generalized anxiety disorder

Eu413 [X]Other mixed anxiety disorders

Eu41y [X]Other specified anxiety disorders

Eu41z [X]Anxiety disorder, unspecified

E200. Anxiety states

E2000 Anxiety state unspecified

E2001 Panic disorder

E2002 Generalised anxiety disorder

E2004 Chronic anxiety

E2005 Recurrent anxiety

E200z Anxiety state NOS

***Mixed depression and Anxiety diagnoses***

E2003 Anxiety with depression

Eu412 [X]Mixed anxiety and depressive disorder

***Depression symptoms***

1B17. Depressed

1B1U. Symptoms of depression

1BQ.. Loss of capacity for enjoyment

1BT.. Depressed mood

1BU.. Loss of hope for the future

2257. O/E – depressed

***Anxiety symptoms***

1B13. Anxiousness

2258. O/E - anxious

1B12. Nerves, nervousness

R2y2. (D) nervousness

2259. O/E nervous
