# Supplementary material for: Case-finding for common mental disorders of anxiety and depression in primary care: an external validation of routinely collected data
Source: BMC Med Inform Decis Mak. 2016 Mar 15;16:35. doi: 10.1186/s12911-016-0274-7 (PMC4791907; doi:10.1186/s12911-016-0274-7)
Supplement: Additional file 2: — Drug treatment Read Codes V2 used in algorithms. (DOCX 16 kb) [file 12911_2016_274_MOESM2_ESM.docx]

**Drug Treatment Read Codes Version 2 (algorithm included packet/bottle level)**

***Antidepressants***

*d71.. Amitriptyline hydrochloride*

*d72.. Butriptyline - discontinued*

*d73.. Clomipramine hydrochloride*

*d74.. Desipramine hydrochloride*

*d75.. Dosulepin Hydrochloride*

*d76.. Doxepin*

*d77.. Imipramine hydrochloride*

*d78.. Iprindole*

*d79.. Lofepramine*

*d7a.. Maprotiline hydrochloride*

*d7b.. Mianserin hydrochloride*

*d7c.. Nortriptyline*

*d7d.. Protriptyline hydrochloride*

*d7e.. Trazadone hydrochloride*

*d7f.. Trimipramine*

*d7g.. Viloxazine hydrochloride*

*d7h.. Amoxapine*

*d81.. Phenelzine*

*d83.. Isocarboxazid*

*d84.. Tranylcypromine*

*d85.. Moclobemide*

*d91.. Compound Antidepressants A-Z*

*da1.. Flupentixol [Antidepressant]*

*da2.. Tryptophan*

*da3.. Fluvoxamine Maleate*

*da4.. Fluoxetine hydrochloride*

*da5.. Sertraline hydrochloride*

*da6.. Paroxetine hydrochloride*

*da7.. Venlafaxine*

*da9.. Citalopram*

*daA.. Reboxetine*

*daB.. Mirtazapine*

*daC.. Escitalopram*

*daD.. Agomelatine*

*gde.. Duloxetine*

***Hypnotics***

d11.. Chloral hydrate

d12.. Clomethiazole edisylate (hypnotic)

d13.. Dichloralphenazone - discontinued

d14.. Flumtrazepam - discontinued

d15.. Flurazepam

d16.. Loprazolam

d17.. Lormetazepam

d18.. Nitrazepam

d1a.. Temazepam (hynotic)

d1b.. Triazolam - discontinued

d1c.. Triclofos sodium

d1d.. Zopiclone

d1f.. Zolpidem

d1g.. Zaleplon

d1h.. Melatonin

d1i.. Dexmedetomidine

***Anxiolytics***

d21.. Diazepam

d22.. Alprazolam

d23.. Bromazepam

d24.. Chlordiazepoxide

d25.. Chlormezanone

d26.. Clobazam

d27.. Clorazepate dipotassium

d28.. Hydroxyzine hcl (anxiolytic)

d29.. Ketazolam - discontinued

d2a.. Lorazepam (anxiolytic)

d2b.. Medazepam - discontinued

d2c.. Meprobamate

d2d.. Oxazepam

d2f.. Buspirone hydrocholoride

d2g.. Flumazenil
